# The Effect of C-Phycocyanin on Microglia Activation Is Mediated by Toll-like Receptor 4

**DOI:** 10.3390/ijms23031440

**Published:** 2022-01-27

**Authors:** Anna Piovan, Raffaella Filippini, Carla Argentini, Stefano Moro, Pietro Giusti, Morena Zusso

**Affiliations:** Department of Pharmaceutical and Pharmacological Sciences, University of Padua, 35131 Padua, Italy; anna.piovan@unipd.it (A.P.); raffaella.filippini@unipd.it (R.F.); carla.argentini@unipd.it (C.A.); stefano.moro@unipd.it (S.M.); pietro.giusti@unipd.it (P.G.)

**Keywords:** C-phycocyanin, microglia, cytokines, Toll-like receptor 4

## Abstract

The blue-green alga *Spirulina platensis* is rich in phycocyanins, that exhibit a wide range of pharmacological actions. C-phycocyanin (C-PC), in particular, possesses hepatoprotective, nephroprotective, antioxidant, and anticancer effects. Furthermore, several studies have reported both anti- and proinflammatory properties of this pigment. However, the precise mechanism(s) of action of C-PC in these processes remain largely unknown. Therefore, here we explored the C-PC effect in in vitro microglia activation. The effect of C-PC on the expression and release of IL-1β and TNF-α and the activation of NF-κB was examined in primary microglia by real-time PCR, ELISA, and immunofluorescence. Treatment with C-PC up-regulated the expression and release of IL-1β and TNF-α. C-PC also promoted the nuclear translocation of the NF-κB transcription factor. Then, to elucidate the molecular mechanisms for the immunoregulatory function of C-PC, we focused on investigating the role of Toll-like receptor 4 (TLR4). Accordingly, several TLR4 inhibitors have been used. Curcumin, ciprofloxacin, L48H37, and CLI-095 that suppresses specifically TLR4 signaling, blocked IL-1β and TNF-α. Overall, these results indicate the immunomodulatory effect of C-PC in microglia cultures and show for the first time that the molecular mechanism implicated in this effect may involve TLR4 activation.

## 1. Introduction

The blue-green alga *Arthrospira platensis*, commonly known as Spirulina, has long been used as a food supplement mainly due to the high content of proteins (~70% by dry weight), vitamins, minerals, various kinds of amino acids, fibers, and pigments such as chlorophylls, carotenoids, and phycocyanins [1,2]. Apart from the importance as a food additive, currently, Spirulina is extensively studied for its potential for medical and therapeutic applications [2]. A lot of in vitro and in vivo studies have been focused on the antioxidant activity of Spirulina, that is able to activate antioxidant enzymes, scavenge free radicals and protect against lipid peroxidation and DNA damage [3,4,5,6]. Thanks to these activities, Spirulina is considered a promising agent with protective roles against renal, hepatic, cardiovascular, and central nervous system (CNS) diseases [7,8,9]. Spirulina has also immunomodulatory and anti-inflammatory properties. As stimulator of the immune system, Spirulina increases the macrophage phagocytic activity, activates T and B cells, and stimulates the production of antibodies and proinflammatory cytokines, such as interleukin (IL)-1β, IL-2, IL-6, tumor necrosis factor (TNF)-α, and interferon-γ [10,11,12]. Spirulina exhibits also promising anti-inflammatory activities being able to inhibit the expression of inflammatory mediators, such as inducible nitric oxide synthase (iNOS), cyclooxygenase-2 (COX-2), TNF-α, IL-1β, and IL-6 in in vitro and in vivo models of inflammation [12,13,14]. The beneficial effects of Spirulina seem to be mediated by β-carotene, phycobiliproteins, and other vitamins and minerals present in the microalga [15,16]. Among the phycobiliproteins, Spirulina contains phycocyanin and allophycocyanin in a ratio of 10:1 [17]. In particular, C-phycocyanin (C-PC), isolated from the blue-green algae, is a nontoxic and noncarcinogenic water-soluble protein that constitutes up to 15–20% of Spirulina dry weight, and it is commonly used as a food additive, cosmetic colorant, and fluorescent dye [18]. C-PC also exhibits a wide range of pharmacological actions, including hepatoprotective, nephroprotective, antioxidant, and anticancer effects [19,20,21]. Furthermore, different studies have shown either anti- or proinflammatory effects of C-PC. For example, in macrophages and BV-2 microglia cells, C-PC reduced the expression of several inflammatory genes (e.g., iNOS, COX-2, TNF-α, and IL-1β) [22,23]. Conversely, Chen et al. [24] showed that C-PC induced secretion of TNF-α, IL-1β, and IL-6, increased expression of COX-2, and stimulated the phosphorylation of proteins implicated in inflammatory responses, including ERK, JNK, p38 and IκB in murine macrophages. Other studies also reported neuroprotective effects of C-PC and showed that oral administration of C-PC crosses blood brain barrier suggesting its use in neurodegeneration, where oxidative stress and neuroinflammation play a relevant role [25,26].

Neuroinflammation is initiated by microglia, the immune cells of the CNS, that in response to pathological conditions undergo an activation process aimed at CNS protection. Microglia activation is characterized by the classical (M1) and the alternative (M2) phenotype, although there are different opinions about the existence of multiple activation phenotypes for microglia. M1 microglia produce proinflammatory cytokines (e.g., TNF-α, IL-1β, IL-6), chemokines, and other mediators (nitric oxide, oxygen radicals), which contribute to the clearance of pathogens. Furthermore, M1 microglia are also able to perform phagocytosis, antigen presentation, and lymphocyte activation. On the other hand, M2 phenotype is associated with neural survival, suppression of brain damage, and prevention of negative effects of the immune response [27,28,29,30]. Importantly, microglia, like peripheral macrophages, are plastic cells that possess the capacity to change their phenotype during the inflammatory response [31,32]. Microglia become activated following interaction of pathogen- and/or endogenous damage-associated molecular patterns with pathogen recognition receptors, that include NOD-like receptors, C-type lectin receptors, RIG-I-like receptors, and Toll-like receptors (TLRs) [33,34]. Among the TLRs, TLR4, localized on the surface of microglia, is the major receptor for lipopolysaccharide (LPS), which is an important component of the outer membranes of Gram-negative bacteria [35]. Following activation, TLR4 interacts with the two adaptor proteins MyD88 and TRIF. Both MyD88-dependent pathway and TRIF-dependent pathway result in the activation of nuclear factor (NF)-κB, which induces the expression of proinflammatory genes and the production of cytokines [36,37].

Considering the conflicting results on the effect of C-PC in in vitro models of inflammation/neuroinflammation, in this study we explored the effect of C-PC in in vitro microglia activation. Then, with the purpose to clarify the mechanism involved in the observed effects, we found that microglia inflammatory response induced by C-PC was mediated by the activation of TLR4.

## 2. Results

### 2.1. Identification of Noncytotoxic Concentrations of C-Phycocyanin in Microglial Cells

**Figure 1:**
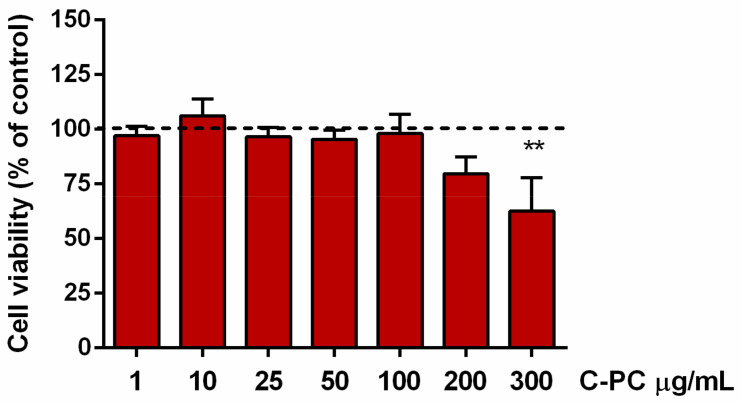
Effect of C-phycocyanin in microglia cell viability. Microglia were cultured overnight in medium containing 10% of serum, which was replaced with serum-free medium before exposure to C-PC (1–300 μg/mL) for 16 h. At the end of incubation, SRB assay was used to measure cell viability. Results are expressed as percentage of cell viability relative to control cells. Data are means ± SEM of 3 independent experiments. ** *p* ˂ 0.01 versus control cells (dashed line). One-way ANOVA followed by Holm–Sidak’s test.

The first experiments were aimed at exploring the safety and identifying the noncytotoxic concentrations of C-PC in microglia. Cultures were incubated with increasing concentrations (1–300 µg/mL) of a commercial preparation of C-PC for 16 h. Cell viability of microglia exposed to C-PC at concentrations higher than 200 µg/mL significantly decreased compared to vehicle treated cells, taken as 100% (Figure 1). Based on these results, concentrations of C-PC used in the following experiments ranged from 1 to 200 µg/mL.

### 2.2. Effect of C-Phycocyanin on Proinflammatory Cytokine Release by Microglia

**Figure 2:**
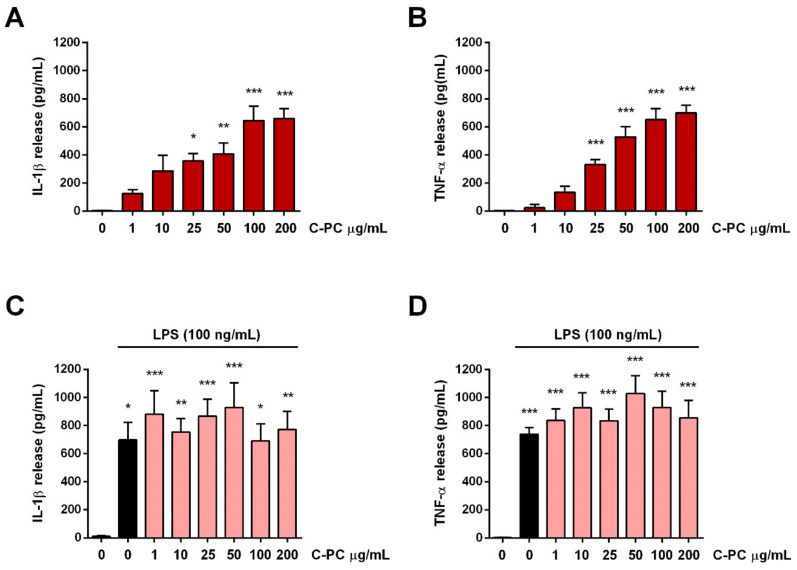
Effect of C-phycocyanin on cytokine release from cortical microglia. Microglia were cultured overnight medium containing 10% of serum, which was replaced with serum-free medium before treatment with C-PC (**A**,**B**) or C-PC + LPS (100 ng/mL) (**C**,**D**). After the exposure to C-PC or LPS for 16 h, supernatants were collected and analyzed for IL-1β (**A**,**C**) and TNF-α (**B**,**D**) content. Data are means ± SEM (*n* = 3 in triplicate). * *p* ˂ 0.05, ** *p* ˂ 0.01, and *** *p* ˂ 0.001 versus control cells. One-way ANOVA followed by Holm–Sidak’s test.

Classical M1 microglia activation is correlated with the production and release of proinflammatory cytokines such as IL-1β and TNF-α, in addition to reactive oxygen species, nitric oxide, and others inflammatory mediators [29]. Therefore, to study the effect of C-PC on M1 microglia activation, cells were treated with noncytotoxic concentrations of C-PC (1–200 µg/mL) in the absence or presence of LPS stimulation, and the release of IL-1β and TNF-α, indicative of M1 activation, was examined. C-PC significantly increased basal levels of both cytokines starting from the concentration of 25 µg/mL (Figure 2A,B). To note that the release of both cytokines induced by the highest concentrations of C-PC tested (100 and 200 µg/mL) was to the same extent as that observed after stimulation with the endotoxin LPS, a potent inducer of M1 microglia activation. Furthermore, pretreatment with C-PC did not influence the release of IL-1β and TNF-α induced by LPS (Figure 2C,D).

**Figure 3:**
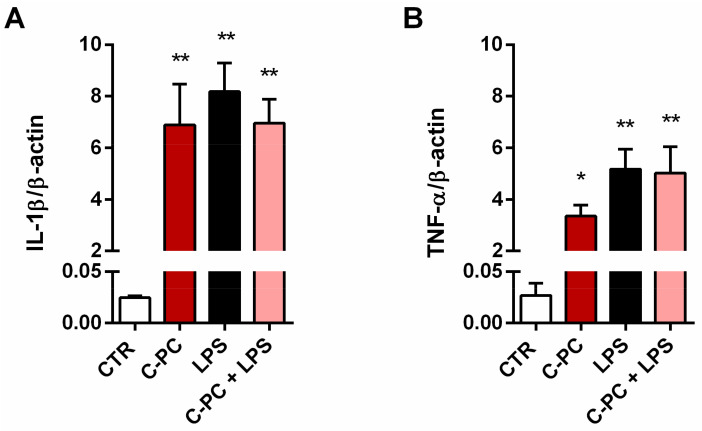
Effect of C-phycocyanin on cytokine production from cortical microglia. Microglia were cultured overnight in medium containing 10% of serum, which was replaced with serum-free medium before treatment with 100 μg/mL C-PC, 100 ng/mL LPS, or their association for 6 h. IL-1β (**A**) and TNF-α (**B**) mRNA levels were quantified by real-time PCR. Data are means ± SEM (*n* = 3 in triplicate). * *p* ˂ 0.05 and ** *p* ˂ 0.01 versus control cells (CTR). One-way ANOVA followed by Holm–Sidak’s test.

To confirm these results, we also explored the effect of C-PC on IL-1β and TNF-α mRNA expression levels. Considering that 100 and 200 µg/mL C-PC affected cytokine release in a similar manner, in the following studies C-PC was used at the concentration of 100 µg/mL. C-PC treatment markedly increased basal levels of cytokine gene expression, without changing the effect of LPS (Figure 3).

### 2.3. Effect of C-Phycocyanin on NF-κB Activation in Microglia

**Figure 4:**
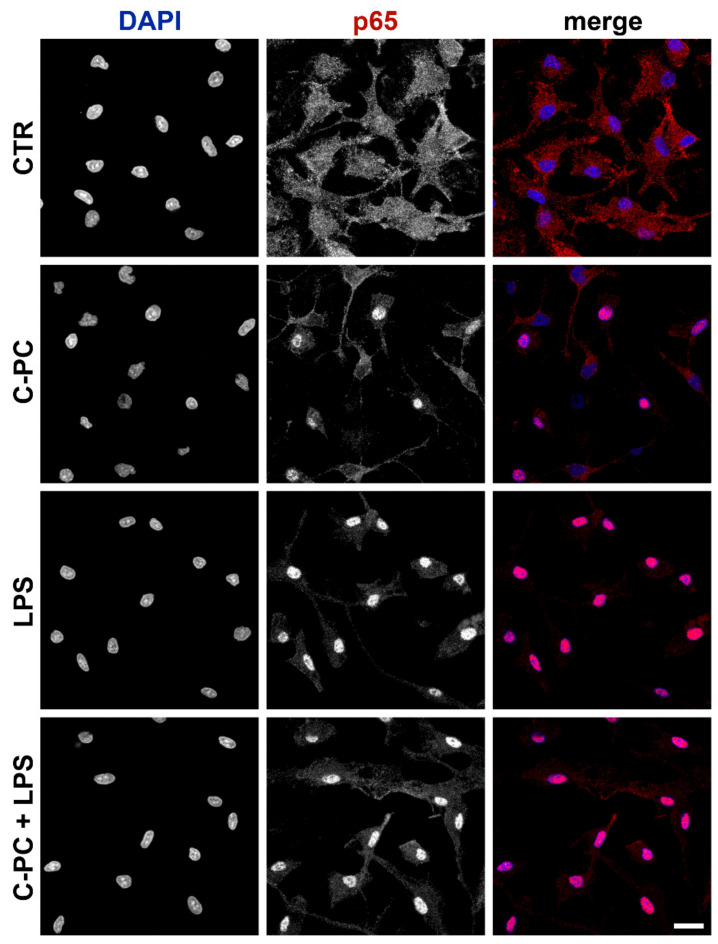
Effect of C-phycocyanin on NF-κB activation in microglia. Microglia were cultured overnight in medium containing 10% of serum, which was replaced with serum-free medium before treatment with 100 μg/mL C-PC ± 100 ng/mL LPS. Cells were then processed for NF-κB p65 immunostaining. Experiments were performed 3 times and representative confocal images showing subcellular localization of p65 are shown. Scale bar, 10 µm.

The transcription factor NF-κB is expressed in the cytoplasm of the majority of cells. The activated NF-κB dimers (p50/p65) translocate to the nucleus and bind to κB site of chromosome to induce transcription of NF-κB targeted genes. NF-κB controls the expression of more than 500 genes, which are involved in inflammatory responses [38,39]. Therefore, immunofluorescence analysis was performed to analyze the effect of C-PC on nuclear translocation of the NF-κB/p65 subunit. Consistent with NF-κB activation, confocal images reveal a pronounced redistribution of p65 subunit from the cytoplasm to the nucleus after treatment with C-PC. Furthermore, as observed for cytokine release, C-PC did not change the effect of LPS (Figure 4). In addition, microglia morphology has not been affected by 90-min exposure to C-PC, LPS, or their association.

### 2.4. Effect of Polymyxin B on Proinflammatory Cytokine Release by Microglia Treated with C-Phycocyanin

**Figure 5:**
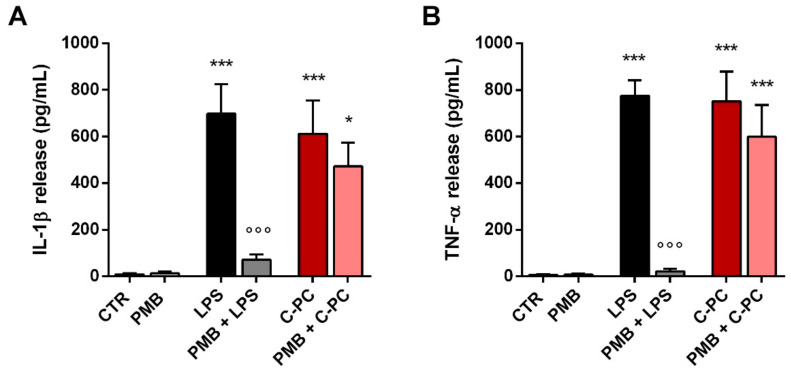
Effect of polymyxin B on cytokine release from cortical microglia. Microglia were cultured overnight in medium containing 10% of serum, which was replaced with serum-free medium before pretreatment with 50 μg/mL polymyxin B (PMB) for 1 h followed by stimulation with LPS (100 ng/mL) or C-PC (100 μg/mL) for 16 h. Supernatant were collected and analyzed for IL-1β (**A**) and TNF-α (**B**) content. Data are means ± SEM (*n* = 4 in triplicate). * *p* ˂ 0.05 and *** *p* ˂ 0.001 versus control cells. °°° *p* ˂ 0.001 versus LPS treatment (black bars). One-way ANOVA followed by Holm–Sidak’s test.

In an attempt to explain the inconsistency between our results and some previous studies that have shown the anti-inflammatory properties of C-PC in macrophage and microglia cultures [23,24], we examined whether C-PC could be contaminated with LPS. Microglia were pretreated with polymyxin B (PMB, 50 µg/mL), a cyclic cationic polypeptide antibiotic able to bind to lipid A and neutralize LPS biological activity, widely used in vitro and in vivo to impede the effects of endotoxin contamination [40,41]. Then, cells were stimulated with 100 ng/mL LPS (used as positive control) or 100 µg/mL C-PC. As expected, PMB markedly abolished LPS-induced release of IL-1β and TNF-α by microglia (Figure 5, gray bars). Conversely, PMB had no effect on the release of both cytokines induced by C-PC (Figure 5, light red bars), proving that C-PC is free from endotoxin contamination.

### 2.5. Effect of Toll-Like Receptor 4 Inhibition on Proinflammatory Cytokine Release from Microglia Treated with C-Phycocyanin

**Figure 6:**
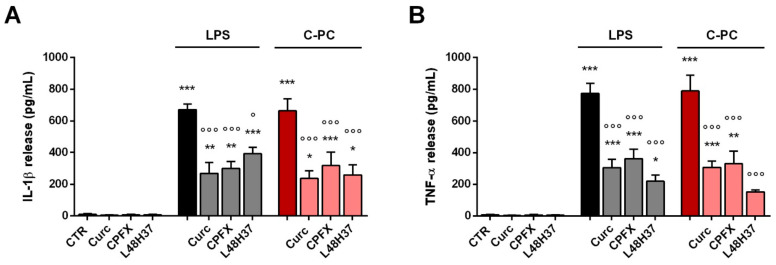
Effect of Toll-like receptor 4 inhibition on cytokine release from cortical microglia. Microglia were cultured overnight in medium containing 10% of serum, which was replaced with serum-free medium before pretreatment with curcumin (Curc, 10 μM), ciprofloxacin (CPFX, 100 μg/mL), or L48H37 (10 μM) for 1 h followed by stimulation with LPS (100 ng/mL) or C-PC (100 μg/mL) for 16 h. Supernatant were collected and analyzed for IL-1β (**A**) and TNF-α (**B**) content. Data are means ± SEM (*n* = 3 in triplicate). * *p* ˂ 0.05, ** *p* ˂ 0.01, and *** *p* ˂ 0.001 versus control cells. ° *p* ˂ 0.05 and °°° *p* ˂ 0.001 versus LPS or C-PC treatment. One-way ANOVA followed by Holm–Sidak’s test.

Next, to determine the target of C-PC at receptor level, we explored whether TLR4 could be required for the proinflammatory effect of C-PC. First, we examined the extracellular region of the receptor complex composed of TLR4 and myeloid differentiation protein 2 (MD-2). To this end, microglia were treated with curcumin, ciprofloxacin, or L48H37, three TLR4 inhibitors that interfere with LPS binding to MD-2 [42,43,44,45]. Curcumin (10 µM), ciprofloxacin (100 µg/mL), and L48H37 (1-ethyl-3,5-bis(3,4,5-trimethoxybenzylidene)piperidin-4-one; 10 µM) reduced the release of IL-1β and TNF-α by microglia stimulated with LPS (used as positive control; Figure 6, gray bars) and C-PC (Figure 6, light red bars). Specifically, the three inhibitors had effects on cytokine release after C-PC stimulation similar to those observed after LPS treatment, suggesting that TLR4 could be the target of the proinflammatory activity of C-PC.

**Figure 7:**
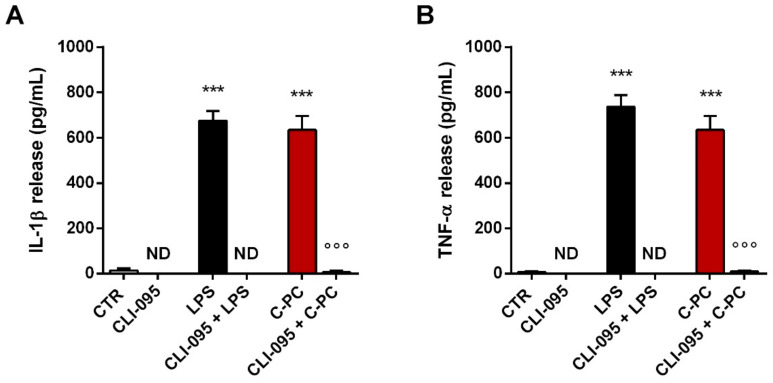
Effect of CLI-095 on cytokine release from cortical microglia. Microglia were cultured overnight in medium containing 10% of serum, which was replaced with serum-free medium before pretreatment with 0.5 µg/mL CLI-095 for 1 h followed by stimulation with LPS (100 ng/mL) or C-PC (100 μg/mL) for 16 h. Supernatant were collected and analyzed for IL-1β (**A**) and TNF-α (**B**) content. Data are means ± SEM (*n* = 3 in triplicate). *** *p* ˂ 0.001 versus control cells and °°° *p* ˂ 0.001 versus C-PC treatment. One-way ANOVA followed by Holm–Sidak’s test. ND, not determined.

Further, we analyzed the role of TLR4 in mediating the effects of C-PC using CLI-095, a cyclohexene derivative that selectively inhibits TLR4 signaling mediated by the receptor intracellular domain [46,47,48]. CLI-095 (0.5 µg/mL) completely reduced the release of IL-1β and TNF-α by microglia stimulated with LPS (Figure 7, gray bars) and C-PC (Figure 7, light red bars), confirming that TLR4 is involved in the inflammatory effect of C-PC.

## 3. Discussion

We have recently shown that an acetone extract from the microalga *Spirulina platensis* reduced the release of proinflammatory cytokines and impeded LPS-triggered neuroinflammation in microglial cells. The studied extract contained chlorophylls, pheophytins and carotenoids that could be implicated in the anti-inflammatory effect observed [14]. Indeed chlorophylls, pheophytins, and carotenoids have been shown to exhibit promising anti-inflammatory activities in numerous experimental models [49,50,51]. *Spirulina platensis* also synthetizes C-PC, a water-soluble pigment, known worldwide as a food additive and cosmetic colorant with potential biological activities and health benefits [52]. Antioxidant and antitumor activities, together with hepatic, renal, cardiovascular, and CNS protective properties of C-PC from *Spirulina platensis* have been extensively shown [19,20,21,22,23,24,25,53,54,55,56,57,58]. Moreover, it should be emphasized that Spirulina and C-PC, in particular, exert anti-inflammatory and immunomodulatory activities by stimulating the production of antibodies and up- or down-regulating the expression of different sets of key cytokines, such as IL-1β, IL-2, IL-4, IL-6, IL-10, and TNF-α [10,11,12,13,14,59].

In the present study we examined the effect of C-PC in the inflammatory phenotype of microglia, the primary innate immune cells of the CNS. Noncytotoxic concentrations of C-PC increased the expression and release of the proinflammatory cytokines IL-1β and TNF-α under basal conditions (i.e., in the absence of an inflammatory stimulus). In addition, C-PC induced translocation to the nucleus of the NF-κB/p65 subunit, indicating the activation of NF-κB signaling, widely implicated in immune responses. These results suggest that C-PC, similarly to LPS, drives microglia into a proinflammatory phenotype, which is characterized by the production of proinflammatory mediators including IL-1β, IL-6, TNF-α, nitric oxide, and reactive oxygen radicals as well as by an increased expression of surface markers such as CD16/32, CD40, CD86, which sustain the inflammatory process [29,60]. Our findings confirm those of Chen et al. [24] that purified C-PC from Spirulina and showed its capability to induce the expression of IL-6, proIL-1β, IL-1β, TNF-α, COX-2 and the phosphorylation of ERK, JNK, p38, and IκB in the murine macrophage cell line J774A.1. However, our results differ from other numerous studies that revealed the anti-inflammatory effect of C-PC in various cell types [19,22,23,57,58], including BV-2 microglial cells. In particular, Chen et al. [23] showed that C-PC prevented the up-regulation of IL-6, TNF-α, iNOS, and COX-2 induced by LPS in BV-2 microglia cells, suggesting that C-PC may contribute to neuroprotection in degenerative disorders in which microglial activation plays a detrimental role. This inconsistency may be partly caused by the difference between primary microglia, used in our study, and the BV-2 cell line. Despite the similarity of BV-2 cells to primary microglia, their use as an alternative model to primary microglia has long been debated. The main idea is that BV-2 cells have almost identical functions as primary microglia, but not to the same extent. For example, upon LPS stimulation, many of the genes induced by BV-2 cells are also up-regulated in primary microglia; however, they are more pronounced in primary microglia compared to the BV-2 cell line [61,62].

C-PC used in this study was extracted and purified from *Spirulina that belongs to the* cyanobacteria group. Cyanobacteria are prokaryotes that contain the basic structure and chemical composition of the cell wall of Gram-negative bacteria, while similarly to eukaryotes they possess a photosynthesis apparatus. Cyanobacterial cell wall contains LPS in the outer membrane layer and LPS comprises 1.6% of the cellular dry weight of Spirulina [63]. For that reason, firstly we verified the presence of LPS in the C-PC preparation used. To this aim, microglia were treated with PMB, a cyclic cationic polypeptide antibiotic produced by *Bacillus polymixa*, that binds and neutralizes LPS of the outer cell membrane of Gram-negative bacteria [64]. PMB did not affect the C-PC-induced microglia inflammatory response, proving that C-PC preparation used in this study was free from LPS contamination and, most important, supporting the direct immunomodulatory effect of C-PC in microglia.

Although C-PC has been widely studied, signaling pathways involved in its biological effects are largely unclear. Thus, here we tried to delineate the target for the immunomodulatory effect of C-PC. Immune response, including that of microglia, initiates with the activation of several classes of pattern recognition receptors, including TLRs. Among them, TLR4 associated with MD-2 is responsible for the initiation of rapid innate immune responses. Binding of ligands, such as LPS, causes dimerization of the extracellular domains and the subsequent recruitment of specific adaptor proteins to the intracellular domains, thus initiating a signaling cascade [32,65]. Previous studies have proposed that inflammatory cascade can be prevented by compounds able to bind to TLR4–MD-2 complex and inhibit its dimerization, required for the activation of downstream signaling pathways [66]. In this context, previous studies, including ours, have shown that curcumin, the major active compound of turmeric, binds directly to the MD-2 pocket, competing with LPS for the same binding site and resulting in the suppression of LPS-induced proinflammatory signaling [42,43]. We also showed that ciprofloxacin, a commonly prescribed antibiotic, can accommodate into the binding pocket of MD-2 occupying a relevant portion of the LPS binding site through the same mechanism of curcumin [44]. Moreover, among the curcumin structural analogues, L48H37 exerts a strong anti-inflammatory activity by targeting MD-2 and inhibiting the formation of TLR4–MD-2–LPS complex [45]. Based on these findings, to explore whether C-PC may target TLR4–MD-2, the receptor complex has been inhibited with curcumin, ciprofloxacin, or L48H37. When microglia were treated with the compounds before stimulation with C-PC, cells showed a suppressed release of IL-1β and TNF-α, suggesting the engagement of TLR4–MD-2 complex in mediating the effect of C-PC. To further support the role of this receptor in mediating the effect of C-PC, microglia were also treated with CLI-095 (also known as TAK-242), a TLR4-specific inhibitor that inhibits TLR4 signaling by binding directly to the intracellular TIR domain of TLR4 [46,47,48]. In our experimental conditions, CLI-095 completely abolished the C-PC-induced release of IL-1β and TNF-α, confirming the role of TLR4 in mediating microglia immune response induced by C-PC. Furthermore, considering that CLI-095 completely blocked the release of both cytokines, the effect of C-PC appears exclusively mediated by TLR4–MD-2 complex.

Even if the binding mode as well as the precise binding site of C-PC on TLR4–MD-2 complex remain to be define, this study helped to clarify the mechanism underlying immunomodulatory activity of C-PC, showing for the first time, to our knowledge, that the signaling through TLR4 can be critically involved. Furthermore, it is noteworthy that in vivo, after oral administration, C-PC is degraded by proteolysis to phycocyanobilin, the linear tetrapyrrole chromophore of C-PC, or to phycocyanobilin-linked peptides [67]. Phycocyanobilin, in particular, possesses anticancer, anti-inflammatory, atheroprotective, nephroprotective, and neuroprotective effects [68,69,70,71,72,73], suggesting that most of the pharmacological actions of C-PC could be ascribed to phycocyanobilin. Therefore, additional in vivo studies will be relevant to definitively prove the functional role of TLR4 in C-PC immunomodulatory effect and to clarify whether phycocyanobilin could contribute to this effect.

## 4. Materials and methods

### 4.1. Reagents

All reagents were from Sigma-Aldrich (Milan, Italy), unless noted otherwise. Tissue culture media, fetal bovine serum (FBS), and antibiotics were purchased from Life Technologies (San Giuliano Milanese, Italy). LPS (Ultra-Pure LPS-EB from *Escherichia coli*, 0111:B4 strain that only activates TLR4) and (6*R*)-6-[*N*-(2-chloro-4-fluorophenyl)sulfamoyl]cyclohex-1-ene-1-carboxylate (CLI-095 or TAK-242) were from InvivoGen (InvivoGen Europe, Toulouse, France). C-PC was purchased from Sigma-Aldrich as a lyophilized powder obtained from *Spirulina platensis* with a 30–50% of protein content and the PC ratios (A_620_/A_280_) > 3.5 [24]. The primary antibody mouse anti-p65 (NF-κB p65, Cat. sc-8008) was from Santa Cruz Biotechnology (Santa Cruz, CA, USA). Alexa Fluor 555 secondary antibody was from Invitrogen (Milan, Italy, Cat. A21422). Enzyme-linked immunosorbent assay (ELISA) kits were obtained from Antigenix America (Huntington Station, NY, USA). Falcon tissue culture plasticwares were purchased from BD Biosciences (SACCO srl, Cadorago (CO), Italy).

### 4.2. Cell Cultures

All experimental procedures were conducted according to national and EU guidelines for animal experiments and were approved by the Institutional Review Board for Animal Research (Organismo Preposto al Benessere Animale, OPBA) of the University of Padua and by the Italian Ministry of Health (protocol number 41451.N.N8P). Microglial cells were isolated from mixed glial cell cultures prepared from cerebral cortices of postnatal day 1 Sprague-Dawley rat pups (CD strain), as previously described [74]. Typically, 7 days after isolation, cultures reached confluence and microglia were recovered by shaking the flasks (200 rpm for 1 h at 37 °C), resuspended in high-glucose Dulbecco’s modified eagle medium (DMEM) supplemented with 2 mM L-glutamine, 10% heat-inactivated FBS, 100 units/mL penicillin, 100 μg/mL streptomycin and 50 μg/mL gentamicin (growth medium), and plated on poly-L-lysine-coated (10 μg/mL) plastic wells at a density of 1.50 × 10^5^ cells/cm^2^. Cells were allowed to adhere for 45 min and then washed to remove nonadhering cells. The procedure used generated microglial cultures of 97% purity, as determined by immunocytochemistry using a primary antibody against ionized calcium binding adaptor molecule 1 (Iba1, 1:800, Wako Chemicals USA Inc., Richmond, VA, USA, Cat. 019-19741), a marker for microglia cell types. Cells were maintained at 37°C in a humidified atmosphere containing 5% CO_2_/95% air.

### 4.3. Cell Viability

The sulforhodamine B (SRB) assay was used to measure microglial cell viability [75,76]. Cells were plated in poly-L-lysine coated 96-well plates (50,000 cells/well) in growth medium containing 10% of serum and allowed to adhere overnight. Growth medium was replaced with serum-free medium 2 h before treatment with increasing concentrations of C-PC for 16 h. At the end of incubation, cells were fixed with cold 10% trichloroacetic acid for 1 h at 4 °C. Then, cells were stained with 0.4% SRB for 30 min at room temperature. Following this step, the protein-bound dye was solubilized with 10 mM Tris base solution. The absorbance was then measured at 570 nm in a microplate reader. Absorbance of vehicle-treated cultures was considered as 100% cell viability.

### 4.4. Cytokine Determination

After the exposure to LPS or C-PC for 16 h, cell supernatants were collected and the content of IL-1β and TNF-α measured using commercially available ELISA kits, according to the manufacturer’s instructions (Antigenix America, Huntington Station, NY, USA). The absolute concentration of cytokines (pg/mL) in the culture medium was calculated from standard curves obtained with known amounts of IL-1β or TNF-α.

### 4.5. Real-Time Polymerase Chain Reaction (Real-Time PCR)

After the exposure to LPS or C-PC for 6 h, total RNA was extracted from cells by QIAzol (Invitrogen), according to the manufacturer’s instructions. The amount and purity of RNA extracted were assessed by RNA 6000 Nano assay in an Agilent BioAnalyser (Thermo Scientific, Milan, Italy). Reverse transcription was performed with SuperScript IV reverse transcriptase (Thermo Fisher Scientific, Milan, Italy). The real-time PCR reaction was performed as described previously [77]. Primers were selected using the NCBI primer designing tool (Primer-Blast), constraining the choice to specific amplification of only one target amplicon for each gene mRNA and to the absence of primer dimers and secondary structures. Primer sequences were: β-actin, 5′-GATCAGCAAGCAGGAGTACGATGA-3′; 5′-GGTGTAAAACGCAGCTCAGTAACA-3’; IL-1β, 5′-CGTCCTCTGTGACTCGTGGG-3′; 5′-ATGGGTCAGACAGCACGAGG-3′; TNF-α, 5′-GCAGGTTCCGTCCCTCTCAT-3′; 5′-TGCCAGTTCCACATCTCGGA-3′. Amounts of amplified product were calculated using linear regression analysis from standard curves, demonstrating amplification efficiencies ranging from 95 to 100%. Dissociation curves were generated for each primer pair, showing single-product amplification. Data were normalized to expression levels of the reference gene β-actin and are presented as specific ratio between the gene of interest and β-actin.

### 4.6. Immunofluorescence

Microglia, grown on coverslips in 24-well plates, were treated for 90 min with 100 μg/mL C-PC, 100 ng/mL LPS, or their association for the analysis of NF-κB activation. Cells were fixed with 4% paraformaldehyde (pH 7.4) for 15 min at room temperature. After blocking nonspecific binding sites with 5% normal goat serum/0.1% Triton X-100 in PBS (blocking solution) for 1 h at room temperature, cells were incubated with the primary antibody anti-p65 (NF-κB p65, 1:500) for 2 h at room temperature. Then, cells were extensively washed with PBS and incubated with the Alexa Fluor 555 secondary antibody (1:1000) for 1 h at room temperature. Both antibodies were diluted in the blocking solution. Negative control omitted the primary antibody. Nuclei were stained with 4,6-diamidino-2-phenylindole (DAPI; 0.1 μg/mL) and coverslips were mounted on microscope slides with Fluoromount-G mounting medium (Fisher Scientific, Milan, Italy) [44]. All images were acquired using a confocal laser-scanning microscope (Zeiss LSM 800; Carl Zeiss AG, Germany). Acquisition settings were kept constant to permit a direct comparison of all images.

### 4.7. Statistical Analysis

Results are given as mean ± SEM. Data were analyzed using GraphPad Prism Software, version 6.0 (GraphPad Software, Inc., San Diego, CA, USA). Statistical analyses were performed by one-way analysis of variance (ANOVA) followed by Holm–Sidak’s post hoc test for multiple comparison. Statistically significant differences were taken at *p* < 0.05.

## Data Availability

The data presented in this study are available on request from the corresponding author.
